# Clinical usefulness of MLCs in robotic radiosurgery systems for prostate SBRT

**DOI:** 10.1002/acm2.12128

**Published:** 2017-07-10

**Authors:** Masashi Tomida, Takeshi Kamomae, Junji Suzuki, Yoichi Ohashi, Yoshiyuki Itoh, Hiroshi Oguchi, Takahito Okuda

**Affiliations:** ^1^ Department of Radiology Toyota Memorial Hospital Toyota Japan; ^2^ Department of Radiological and Medical Laboratory Sciences Nagoya University Graduate School of Medicine Nagoya Japan; ^3^ Department of Therapeutic Radiology Nagoya University Graduate School of Medicine Nagoya Japan; ^4^ Department of Quality Management for Radiotherapy Toyota Memorial Hospital Toyota Japan

**Keywords:** multileaf collimator, patient‐specific quality assurance, prostate cancer, robotic radiosurgery system, stereotactic body radiation therapy

## Abstract

Stereotactic body radiation therapy (SBRT) using recently introduced multileaf collimators (MLC) is preferred over circular collimators in the treatment of localized prostate cancer. The objective of this study was to assess the clinical usefulness of MLCs in prostate SBRT by comparing the effectiveness of treatment plans using fixed collimators, variable collimators, and MLCs and by ensuring delivery quality assurance (DQA) for each. For each patient who underwent conventional radiation therapy for localized prostate cancer, mock SBRT plans were created using a fixed collimator, a variable collimator, and an MLC. The total MUs, treatment times, and dose**–**volume histograms of the planning target volumes and organs at risk for each treatment plan were compared. For DQA, a phantom with a radiochromic film or an ionization chamber was irradiated in each plan. We performed gamma‐index analysis to evaluate the consistency between the measured and calculated doses. The MLC‐based plans had an ~27% lower average total MU than the plans involving other collimators. Moreover, the average estimated treatment time for the MLC plan was 31% and 20% shorter than that for the fixed and variable collimator plans respectively. The gamma‐index passing rate in the DQA using film measurements was slightly lower for the MLC than for the other collimators. The DQA results acquired using the ionization chamber showed that the discrepancies between the measured and calculated doses were within 3% in all cases. The results reinforce the usefulness of MLCs in robotic radiosurgery for prostrate SBRT treatment planning; most notably, the total MU and treatment time were both reduced compared to the cases using other types of collimators. Moreover, although the DQA results based on film dosimetry yielded a slightly lower gamma‐index passing rate for the MLC than for the other collimators, the MLC accuracy was determined to be sufficient for clinical use.

## 1 INTRODUCTION

Radiation therapy is a standard type of treatment for prostate cancer.^1^, ^2^ By employing innovative techniques such as intensity‐modulated radiation therapy (IMRT), highly conformal dose distributions, and steep dose gradients can be achieved.^3^ Consequently, dose escalation can be performed safely without increasing the normal tissue toxicity.^4^

Recently, stereotactic body radiation therapy (SBRT) has been used clinically for prostate cancer treatment because the *α*/*β*‐ratio of the prostate is lower than those of the surrounding late‐responding normal tissues.^5^, ^6^ Most of the data reported for prostate SBRT have been acquired using CyberKnife (Accuray, Inc., Sunnyvale, CA, USA), which is the only commercially available robotic radiosurgery system.^6^ Those results have demonstrated the safety and efficacy of SBRT, making it a practical treatment method. CyberKnife has a compact 6 MV X‐band linear accelerator that is mounted on a six‐joint industrial robotic arm and includes an advanced image guidance system. This system provides a large number of noncoplanar, nonisocentric beams and ensures flexibility in beam pattern generation, allowing it to produce highly conformal dose distributions to target volumes.^7^

Until recently, CyberKnife was only used in circular therapeutic fields collimated by fixed collimators and Iris Variable Aperture Collimators (Accuray, Inc., Sunnyvale, CA, USA). However, a new series of systems equipped with multileaf collimators (MLCs) has proven to be clinically useful.^8^, ^9^ Most reports have demonstrated the usefulness of MLCs relative to other existing collimators in treatment planning, e.g., by comparing dose**–**volume histograms and treatment times. Moreover, the report of American Association of Physicists in Medicine (AAPM) Task Group 135 recommends performing delivery quality assurance (DQA) tests using high‐spatial‐resolution detectors,^10^ and GafChromic EBT film (International Specialty Products, Wayne, NJ, USA) has been demonstrated to be suitable for CyberKnife dosimetry.^11^ Nevertheless, to our knowledge, a comparison of the DQA results for three different types of collimators has never been published. In SBRT, the discrepancies between the delivered and calculated dose distributions could substantially impact the probability of tumor control since the treatment fraction number is lower than those of conventional radiation therapy techniques.^12^ In addition, the prostate is anatomically located next to the rectum, bladder, and gastrointestinal duct. We believe that the required accuracy of prostate SBRT should either equal or surpass that of other sites such as intracranial and lung lesions. Therefore, the objective of this study was to assess the clinical usefulness of robotic radiosurgery systems equipped with MLCs in prostate SBRT by comparing the treatment planning results and the DQA results with those obtained using other types of collimators.

## 2 MATERIALS AND METHODS

### 2.A Fundamental comparison of measurements and calculations

**Figure 1:**
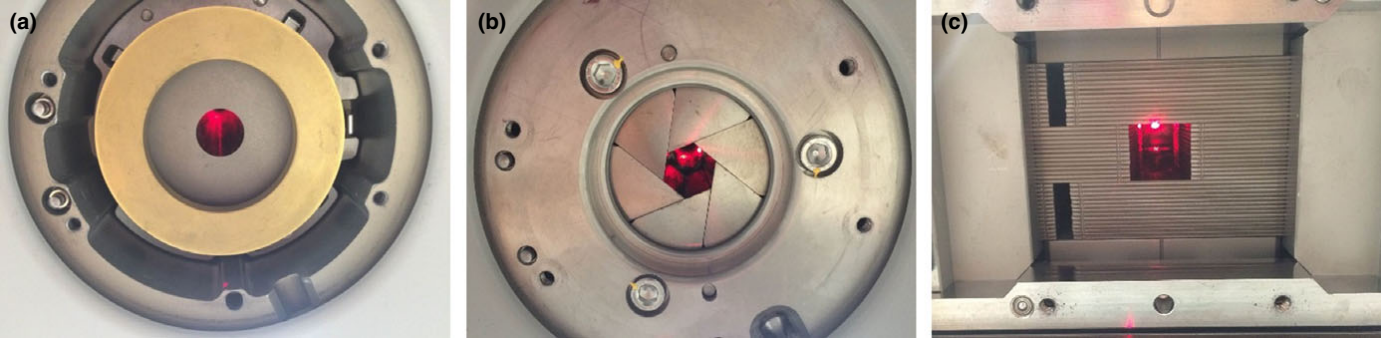
Images of (a) fixed collimator, (b) variable collimator, and (c) MLC.

We compared the measured and calculated doses in a single beam produced by a CyberKnife M6 with three different types of collimators (Fig. 1). Firstly, the differences between the off‐center ratios (OCRs) were evaluated using the commissioning tools of a MultiPlan 5.1.3 Treatment Planning System (Accuray, Inc., Sunnyvale, CA, USA). The commissioning tools can be employed to output the measured and calculated doses corresponding to a single beam at several depths in water. In the beam profile examination in this study, we determined the OCRs at depths of 15 mm and 100 mm with variously sized single beams formed by the fixed collimator, variable collimator, and MLC. The source–axis distance (SAD) was 800 mm, and the fields produced by the fixed and variable collimators at this SAD had diameters of 7.5 mm, 12.5 mm, 30 mm, and 60 mm, while those produced by the MLC had dimensions of 7.6 mm × 7.5 mm, 12.6 mm × 12.5 mm, 32.6 mm × 32.5 mm, and 62.6 mm × 62.5 mm. The ranges of the OCRs were evaluated as ±30 mm in fields with areas of less than 200 mm^2^ and ±50 mm in fields with areas larger than 200 mm^2^. The regions used to compare the OCRs were divided according to the doses delivered to them: the inside/penumbra region was defined as that with more than 20% of the central axis dose, while the outside region was defined as that with less than 20% of the central axis dose.

**Figure 2:**
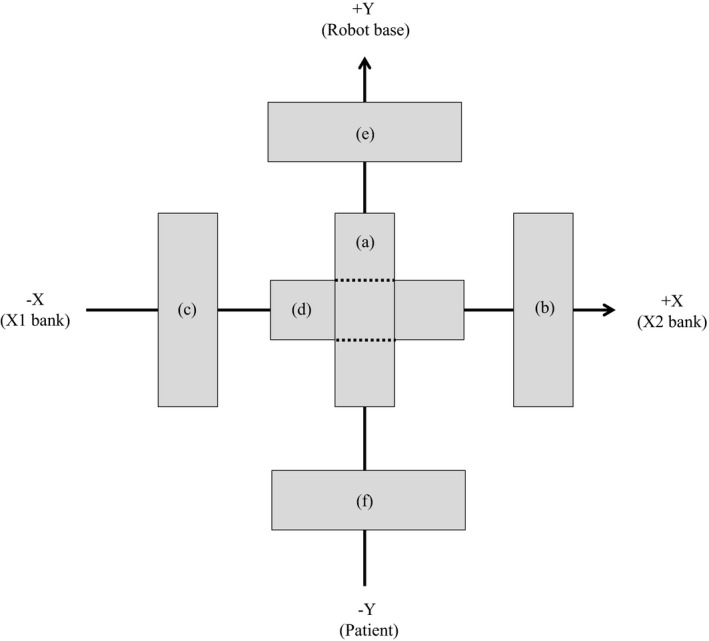
Geometrical arrangement of radiation fields for penumbra width comparison with MLC. The penumbrae were oriented and located as follows: (a) along the *X*‐axis and centered, (b) along the *X*‐axis and shifted by 30 mm in the +X direction, (c) along the *X*‐axis and shifted by 30 mm in the −X direction, (d) along the *Y*‐axis and centered, (e) along the *Y*‐axis and shifted by 30 mm in the +Y direction, and (f) along the *Y*‐axis and shifted by 30 mm in the −Y direction.

Next, we compared the measured and calculated penumbra widths corresponding to a single radiation beam collimated by the MLC. The films were exposed to 500 MU, and the analysis methods and equipment were the same as those employed in the subsequent DQA. The source–surface distance was 800 mm, and the films were inserted at a depth of 90 mm. The radiation fields formed by the MLC are depicted in Fig. 2 and had the following dimensions and locations: (a) 17.4 mm × 47.5 mm and centered, (b) 17.4 mm × 47.5 mm and shifted 30 mm from the center in the +X direction, (c) 17.4 mm × 47.5 mm and shifted 30 mm from the center in the −X direction, (d) 47.4 mm × 17.5 mm and centered, (e) 47.4 mm × 17.5 mm and shifted 30 mm from the center in the +Y direction, and (f) 47.4 mm × 17.5 mm and shifted 30 mm from the center in the −Y direction. We evaluated the penumbrae along the X direction in geometry (a)–(c) and along the Y direction in geometry (d)–(f). The widths of the penumbrae were defined such that each contained 20%–80% of the maximum dose of the corresponding field. In addition, the penumbrae calculated for a 15‐mm‐diameter field defined by the fixed and variable collimators were compared with those measured using the same collimators.

### 2.B Patient characteristics

This study involved 10 patients with biopsy‐proven prostate carcinoma (T1c–T3a), without any nodal and distant metastases (N0, M0), who underwent moderate hypofractionated radiation therapy (70 Gy/28 fr) with CyberKnife between February and August 2016. Three gold markers were implanted transperineally into the prostate under transrectal ultrasound guidance by the urologists. The median patient age was 66 yr (range, 54–79 yr). This study was approved by the Institutional Review Board of Toyota Memorial Hospital and all of the patients provided written informed consent.

### 2.C Treatment planning

Computed tomography (CT) scans for treatment planning were conducted using an Optima CT580W (GE Medical Systems, Waukesha, WI, USA), and the scan parameters were as follows: slice thickness, 1.25 mm; field of view, 500 mm (512 × 512 pixels); X‐ray tube voltage, 120 kV; and X‐ray tube current, 420 mA. One radiation oncologist delineated the target and critical organs in the acquired CT images, which were then fused with magnetic resonance images. The clinical target volume (CTV) was defined as the whole prostate only or as the whole prostate plus the proximal seminal vesicles, depending on the stage of the disease.^13^ To obtain each planning target volume (PTV), the CTV was expanded 3 mm posteriorly and 5 mm in all of the other dimensions. We then created mock hypofractionated radiation therapy plans. The prescription dose was 36.25 Gy in 95% of the PTV and was delivered in five fractions, as in extreme hypofractionated radiation therapy. The parameters, such as the PTV margin, prescription dose, and treatment fraction number, were identical to those used in the prospective phase II trials conducted by King et al.^5^ Moreover, the dose**–**volume histogram (DVH) goals used for the critical tissues, which are described hereinafter, were set in accordance with those reported by King et al.^5^ The rectum DVH goals were V50% < 50% (i.e., the volume receiving 50% of the prescribed dose was <50%), V80% < 20%, V90% < 10%, and V100% < 5%. The bladder DVH goals were V50% < 40% and V100% < 10%. The femoral head dose‐volume goal was V40% < 5%. The prostate SBRT plans were created using a MultiPlan 5.1.3 Treatment Planning System for each type of collimator (the fixed collimator, variable collimator, and so‐called InCise MLC) for the CyberKnife M6. The fixed collimator plans were developed for two differently sized collimators with diameters between 15 mm and 35 mm, and variable collimator plans were developed for three differently sized collimators with diameters between 12.5 mm and 40 mm. All treatment plans were generated by a medical physicist. The “sequential optimization” method described in detail by Descovich et al.^14^ was used for plan generation. We created the planning optimization script for each collimator type. The treatment plans were performed according to the script during initial optimization, and the optimization parameters were then adjusted until the required dose constraints were met. The MLC plans were generated using the conformal avoidance method with the corresponding radiation fields having eroded, perimeter, and random shapes. Ray‐tracing dose calculation algorithms were employed to develop the fixed and variable collimator plans,^15^ while a finite‐sized pencil beam (FSPB) algorithm was used to generate the MLC plans since ray‐tracing algorithms could not be applied in those cases.

### 2.D DQA

For the DQA, we used an I'mRT Phantom (IBA Dosimetry, Schwarzenbruck, Germany) that was composed of water‐equivalent RW3 material with radio‐opaque markers for tracking and acquired the CT images with the following parameters: slice thickness, 0.625 mm; field of view, 320 mm (512 × 512 pixels); X‐ray tube voltage, 120 kV; and X‐ray tube current, 400 mA. We inserted GafChromic EBT3 films (Ashland Specialty Ingredients, Wayne, NJ, USA) into the axial, sagittal, and coronal planes of the phantom to acquire the dose distribution measurements. In the treatment planning system (TPS), we oriented the beam arrangement on the phantom so that the center of the PTV aligned with that of the film. The calculation grid in the TPS had dimensions of 0.625 mm × 0.625 mm × 0.625 mm. In addition, the MUs were reduced by half in the phantom irradiation plans compared to their values in the patient plans to avoid saturation of the films, which were highly sensitive.

Absolute dose measurements were obtained for the same I'mRT phantom using an Exradin A16 ionization chamber (Standard Imaging Inc., Middleton, WI, USA) with a collecting volume of 0.007 cm^3^. The ionization chamber was placed at the center of the phantom for all of the measurements. The doses calculated by the TPS that were compared with measured doses were the average doses in the collecting volume of the ionization chamber. Unlike in the film dosimetry process, the MUs of the phantom plans were not reduced in the ionization chamber dosimetry investigation.

### 2.E Irradiated film analysis

To establish the dose**–**response curve of the GafChromic EBT3 film, irradiation was performed using the 6 MV photon beam of CyberKnife at the center of a 60‐mm‐diameter field with the fixed collimator. In total, 25 films were irradiated with doses of 0–750 cGy at a depth of 100 mm and a source–film distance of 800 mm in a water‐equivalent phantom (Tough Water; Kyoto Kagaku Co., Kyoto, Japan). The doses along the dose**–**response curve of the film were calibrated against those measured using an Exradin A12S ionization chamber (Standard Imaging Inc., Middleton, WI, USA) with a collecting volume of 0.24 cm^3^.

The irradiated films were scanned using an Epson ES‐G11000 flatbed scanner (Seiko Epson Corp., Nagano, Japan), where the Epson Scan driver, Epson Scan, was operated in the 48‐bit RGB mode via a DD‐System (R‐TECH Inc., Tokyo, Japan) with a resolution of 150 dots per inch. The digitized data in the red color channel were analyzed using the DD‐System.^16^, ^17^ The films were allowed to set for 24 hr before scanning to avoid the effects of postexposure density growth.^18^, ^19^ In the DQA analysis, the doses were normalized, so as to match the calculated dose at the center of the volume of interest in the PTV. Therefore, this analysis was an evaluation of the consistency between measured and calculated values of relative dose distributions. As irradiated films were marked to indicate the position of the in‐house umbonate phantom, we aligned the position in the film analysis by matching the markings made for the umbos during measurements and calculations. Gamma‐index analysis 20 was used to compare the measurements and calculations, by applying a criterion of 3% local pixel dose difference (LPDD)/2 mm distance‐to‐agreement (DTA) and a threshold of 30% of the maximum dose. This criterion was determined based on previous studies,^21^, ^22^, ^23^ and the threshold was set according to the dose constraints of the critical organs.

### 2.F Statistical analysis

Statistical analysis was performed by using IBM SPSS Statistics 24 (IBM SPSS Inc., Chicago, IL, USA). The Kruskal**–**Wallis test was conducted to compare the effects of using the different types of collimators on the treatment planning and DQA results. The null hypothesis was that the treatment planning and DQA results would not differ between the different types of collimators. A significance level of 0.05 was used to reject the null hypothesis.

## 3 RESULTS

The beam commissioning results presented in Table 1 reveal that the average dose discrepancy between the measured and calculated doses is larger for the MLC than for the other types of collimators. For the inside/penumbra region, the dose discrepancy resulting from using the MLC is more than 3% in small fields, while those corresponding to the fixed and variable collimators are within 0.1%, regardless of the collimator size. Moreover, in the outside region, the average dose discrepancies for the fixed collimator, variable collimator, and MLC are within 0.1%, 0.1%, and 0.4% respectively.

**Table 1:** Average discrepancy between measured and calculated doses in single beam

Depth (mm)	Field size^a^ (mm)	Fixed collimator (%)	Variable collimator (%)	MLC (%)	
				Crossline	Inline
(a) Inside/penumbra region					
15	7.5 (7.6 × 7.5)	0.03	0.03	4.33	2.38
	12.5 (12.6 × 12.5)	0.03	0.01	2.04	0.61
	30.0 (32.6 × 32.5)	0.05	0.05	1.01	0.53
	60.0 (62.6 × 62.5)	0.03	0.02	0.57	0.30
100	7.5 (7.6 × 7.5)	0.05	0.02	3.56	2.10
	12.5 (12.6 × 12.5)	0.03	0.02	1.66	0.76
	30.0 (32.6 × 32.5)	0.04	0.04	0.89	0.61
	60.0 (62.6 × 62.5)	0.04	0.02	0.57	0.42
(b) Outside region					
15	7.5 (7.6 × 7.5)	0.08	0.06	0.14	0.33
	12.5 (12.6 × 12.5)	0.04	0.07	0.11	0.32
	30.0 (32.6 × 32.5)	0.01	0.01	0.11	0.02
	60.0 (62.6 × 62.5)	0.04	0.02	0.16	0.28
100	7.5 (7.6 × 7.5)	0.02	0.03	0.10	0.28
	12.5 (12.6 × 12.5)	0.03	0.01	0.11	0.26
	30.0 (32.6 × 32.5)	0.03	0.02	0.14	0.11
	60.0 (62.6 × 62.5)	0.06	0.00	0.17	0.18

a The field sizes are represented as diameters for the fixed and variable collimators (outside the parentheses) and as dimensions in the X and Y directions for the MLC (inside the parentheses).

The measured and calculated penumbra widths for the MLC are presented in Table 2. The average discrepancies in the X‐direction (parallel to the MLC's direction of motion) and Y‐direction (perpendicular to the MLC's direction of motion) are 0.2 mm (maximum, 0.3 mm) and 0.3 mm (maximum, 0.7 mm) respectively. Meanwhile, the measured and calculated penumbra widths for the fixed collimator were found to be 3.7 mm and 3.7 mm on one side (corresponding to +X side of MLC in Fig. 2) and 3.7 mm and 3.8 mm on the other side (corresponding to −X side of MLC in Fig. 2) respectively. Likewise, the measured and calculated penumbra widths for the variable collimator were determined to be 3.7 mm and 3.6 mm on one side (corresponding to +X side of MLC in Fig. 2) and 3.6 mm and 3.6 mm on the other side (corresponding to −X side of MLC in Fig. 2). For the circular collimator, the penumbra width discrepancy was at most 0.1 mm.

**Table 2:** Measured and calculated penumbra widths for rectangular fields formed by MLC

	Penumbra width (mm)					
	(c) Shifted 30 mm in the −X direction		(a) Centered		(b) Shifted 30 mm in the +X direction	
	Measurement	Calculation	Measurement	Calculation	Measurement	Calculation
−X direction	4.1	4.2	3.7	3.9	4.0	3.8
+X direction	4.1	3.8	3.7	4.0	4.0	4.2

	Penumbra width (mm)					
	(f) Shifted 30 mm in the −Y direction		(d) Centered		(e) Shifted 30 mm in the +Y direction	
	Measurement	Calculation	Measurement	Calculation	Measurement	Calculation
−Y side	3.4	4.1	3.6	3.8	3.3	3.7
+Y side	3.9	3.7	4.0	3.8	4.1	4.1

The parameters of the prostate SBRT plans developed using each type of collimator are summarized in Table 3. The average number of beams in the MLC plans is 28% and 32% lower than those in the fixed and variable collimator plans respectively. The average total MU of the MLC plans is about 27% lower than those of the others. Moreover, the average estimated treatment time of the MLC plans is 31% and 20% shorter than those of the fixed and variable collimator plans respectively. All three of these parameters differ significantly between the MLC plans and the other collimator plans.

**Table 3:** Parameters of plans developed using each type of collimator

Collimator	Number of beams	Total MU (10^3^)	Estimated treatment time (min)
Fixed	177 ± 31*	36.2 ± 2.8*	46 ± 4*
Variable	187 ± 34**	36.4 ± 2.3**	40 ± 3**
MLC	127 ± 35* ^,^ **	26.3 ± 3.0* ^,^ **	32 ± 4* ^,^ **
Statistics	*P* < 0.05* ^,^ **	*P* < 0.05* ^,^ **	*P* < 0.05* ^,^ **

^a^In this table, *indicates that there is a significant difference between the fixed collimator and MLC results. Likewise, **indicates that there is a significant difference between the variable collimator and MLC results.

The parameters of the prostate SBRT DVHs corresponding to each type of collimator are summarized in Table 4. All of the treatment plans developed using each collimator type met the critical organ dose constraint requirements set forth in a previous report.^5^ In the MLC plans, V50% for the bladder is about 30% lower than it is in the circular collimator plans, and this difference is statistically significant. However, none of the other DVH indices differs significantly among the collimator types.

**Table 4:** Parameters of DVHs corresponding to plans developed using each type of collimator

Collimator	Coverage (%)	Prescribed isodose line (%)	Rectum (%)		Bladder (%)		Femoral head (%)
			V50%	V100%	V50%	V100%	V40%
Fixed	95.0 ± 0.2	82.5 ± 2.1	28.8 ± 10.6	1.5 ± 0.8	24.9 ± 7.4*	2.3 ± 1.4	n/a
Variable	95.1 ± 0.5	83.6 ± 3.2	31.3 ± 10.9	1.2 ± 0.7	25.7 ± 7.8**	2.2 ± 1.4	n/a
MLC	95.4 ± 0.5	82.4 ± 2.5	29.2 ± 10.0	1.4 ± 0.8	17.4 ± 7.6* ^,^ **	2.3 ± 1.3	n/a
Statistics	n.s.	n.s.	n.s.	n.s.	*P* < 0.05* ^,^ **	n.s.	n/a

^a^In this table, *indicates that there is a significant difference between fixed collimator and MLC results. Likewise, **indicates that there is a significant difference between the variable collimator and MLC results.

n.s., not significant; n/a, not applicable.

**Figure 3:**
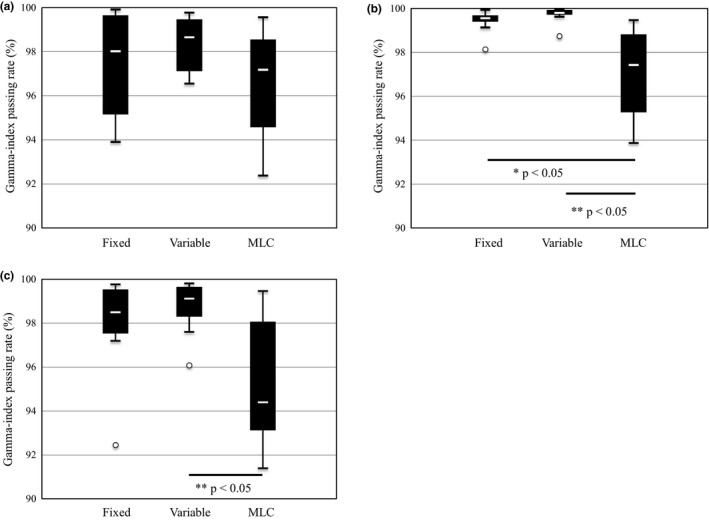
Boxplots of gamma‐index passing rates in the (a) axial, (b) sagittal, and (c) coronal planes. In this figure, * indicates that there is a significant difference between the fixed collimator and MLC results. Likewise, ** indicates that there is a significant difference between the variable collimator and MLC results.

The DQA results obtained in each plane by performing film measurements of the dose distributions are summarized in Table 5 and illustrated by the boxplots in Figs. 3a–3c. The average gamma‐index passing rates in the axial plane are 97.4% ± 2.4%, 98.4% ± 1.2%, and 96.5% ± 2.7% for the fixed collimator, variable collimator, and MLC plans, respectively. Likewise, those in the sagittal plane are 99.5% ± 0.5%, 99.7% ± 0.4%, and 97.1% ± 2.0%, and those in the coronal plane are 98.0% ± 2.2%, 98.7% ± 1.2%, and 95.2% ± 2.8%. Furthermore, the passing rates in the axial plane for the different types of collimators do not differ significantly (*P* = 0.23). However, the MLC results differ significantly from those of the other collimators in the sagittal plane (*P* < 0.05), while a significant difference only exists between the MLC and variable collimator results in the coronal plane (*P* < 0.05). However, the 3% LPDD and 2 mm DTA gamma‐index analysis yielded a pass rate greater than 90% for each collimator type in each plane.

**Table 5:** Gamma‐index passing rates for each patient

Patient #	Gamma‐index passing rate (%)								
	Fixed collimator			Variable collimator			MLC		
	Axial	Sagittal	Coronal	Axial	Sagittal	Coronal	Axial	Sagittal	Coronal
1	98.3	99.9	99.8	99.6	100.0	98.3	94.6	99.5	99.5
2	99.7	99.4	99.1	99.5	99.7	99.5	96.2	95.9	93.8
3	99.9	99.7	99.8	98.4	99.7	99.7	93.1	95.0	96.4
4	99.9	99.6	99.5	99.8	99.8	99.7	92.4	93.9	93.1
5	99.4	99.6	98.2	98.6	99.9	99.8	98.1	99.3	94.9
6	95.2	99.1	97.5	98.7	99.9	99.2	98.6	98.2	98.1
7	93.9	99.6	98.0	96.6	99.6	99.1	99.6	95.3	92.3
8	94.7	98.1	92.5	98.7	99.8	96.1	98.5	98.8	91.4
9	95.2	99.6	97.2	97.1	100.0	98.3	99.6	98.2	98.3
10	97.8	99.9	98.8	96.9	98.7	97.6	94.6	96.7	93.9

**Figure 4:**
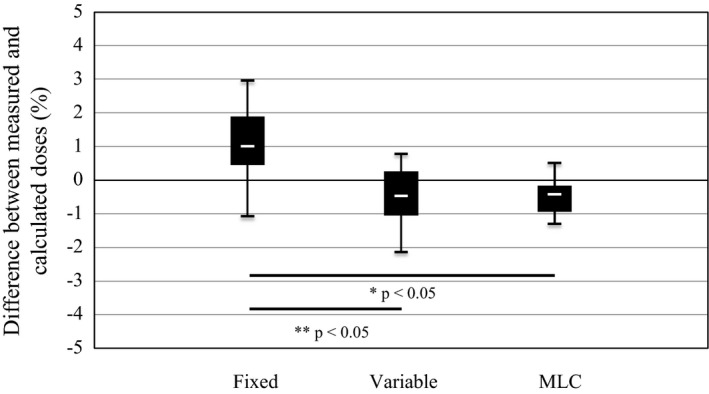
Differences between doses measured using ionization chamber and calculated doses for each collimator type. In this figure, * indicates that there is a significant difference between the fixed collimator and MLC results. Likewise, ** indicates that there is a significant difference between the fixed collimator and variable collimator results.

Figure 4 presents the absolute dose measurements obtained using the ionization chamber. The average dose differences between the measured and calculated doses are 1.1% ± 1.2%, −0.5% ± 0.9%, and −0.5% ± 0.5% with for the fixed collimator, variable collimator, and MLC, respectively. For each collimator type, the difference between the measured and calculated doses is within 3%. However, the fixed collimator results are significantly different from those obtained using the other collimator types (*P* < 0.05).

## 4 DISCUSSION

In treatment planning, the prostrate SBRT dose constraint proposed by King et al. was satisfied in each plan generated using each type of collimator. The total MUs in the MLC plans were about 27% less than those in the fixed and variable collimator plans. In addition, the average estimated treatment time was about 31% and 20% shorter in the MLC plans than it was in the fixed and variable collimator plans respectively. Kathriarachchi et al. reported that the prostate SBRT plans they developed for a CyberKnife M6 with an InCise MLC had total MUs and estimated treatment times reduced by 42% and 36% relative to those for variable collimator plans.^9^ Moreover, McGuinness et al. reported that their MLC plans had average total MUs and treatment times reduced by 40% and almost 50% compared to those of circular collimator plans.^8^ Although the reduction rates in this study were less than those obtained in the abovementioned previous studies, it is difficult to compare the results fairly because those values depend on the specific parameters of both the MLC and circular collimator plans. For example, the average total MU of the circular collimator plans developed by McGuinness et al. was 39.7 × 10^3^ MU, whereas that of the fixed collimator plans in this study was 36.2 × 10^3^ MU. Therefore, if we had created circular plans with larger MUs, the MU reductions achieved by the MLC plans would have been greater than the present rate of 27%.

The DVH results revealed that V50% for the bladder was significantly lower in the MLC plans than in the other collimator plans. Similarly, it was previously reported that MLC plans could yield generalized equivalent uniform doses for the bladder that were lower than those obtained using plans based on other types of collimators.^8^, ^9^ On the other hand, the rectum dose did not differ significantly between the different collimator types, as was found previously.^8^, ^9^ The CyberKnife workspace extends from lateral to anterior, to accommodate patients in supine positions, and the ability to arrange beams below the couch is limited.^24^ Therefore, we assumed that the doses received by the bladder, which is located ventral to the prostate anatomically, could be reduced in the MLC plans relative to those in the circular collimator plans because of the MU reduction and the ability to fit the radiation fields to irregular PTV shapes. However, since the rectum is mostly attached to the prostate and has a relatively small cross‐section, we expected only slight differences among the doses received by the rectum in the treatment plans developed using the different types of collimators. The results of this study indicate that performing treatment planning using an MLC provides substantial improvements compared to when circular collimators are used; specifically, the treatment time and total MUs can be significantly reduced, while achieving an equivalent or superior dose distribution. Due to the formation of complicated radiation fields because of using MLCs, the required beam numbers in the MLC plans were smaller than the ones in other collimator plans. Consequently, the reduction in the beam number could lead to lower MUs and shorter treatment times, provided the PTV volume and shape were invariant. These advantages would reduce the burdens on patients and improve the treatment efficiency.

The patient‐specific DQA results obtained by performing film dosimetry and gamma‐index analysis were slightly poorer for the MLC than they were for the other collimator types. We expect that there are two likely causes of these results: the reproducibility of the leaf position of the MLC and the dependence of the MLC beam modeling accuracy on the TPS. Concerning the collimator position reproducibility, a variable collimator is designed to achieve an aperture reproducibility of ≤0.2 mm at a SAD of 800 mm.^25^ Meanwhile, Fürweger et al. demonstrated using Bayouth tests that the average leaf position offset resulting from using a Cyberknife M6 with an InCise MLC was ≤0.5 mm.^24^ Regarding the second factor affecting the DQA results, the beam modeling accuracy in the MLC beams was found to be inferior to those in the beams based on the other collimators, since the beam commissioning results revealed dose discrepancies of greater than 3% between the measured and calculated doses in the MLC beams. Moreover, although circular collimators have radiation fields that are always symmetric about the central beam axis for all beams, MLCs make effective use of off‐axis fields. Only two directional OCRs (crossline and inline) and two diagonal OCRs are required to model beams in TPSs when MLCs are used. Therefore, the dose calculation precision could have deteriorated because the off‐axis beams were frequently calculated by interpolation.^26^

The accuracy of beam modeling in TPSs when MLCs are employed can be analyzed in further detail by considering the corresponding penumbrae. The measured widths of the penumbrae formed by the MLC leaf tips (along the *X*‐axis) were lower near the center of the MLC (3.7 mm) than they were toward its sides (4.0–4.1 mm). On the other hand, the penumbrae formed by the leaf sides (along the *Y*‐axis) were wider on the +Y sides of the leaves than they were on the −Y sides. This asymmetry was regarded as resulting from the leaf bank tilt.^24^ These penumbra width results are similar to those reported by Fürweger et al.^24^ Furthermore, although the discrepancies between the measured and calculated widths of the *X*‐axis penumbrae were relatively small (maximum, 0.3 mm), those between the measured and calculated widths of the *Y*‐axis penumbrae were large (maximum, 0.7 mm) when the MLC was used. On the other hand, the discrepancies resulting from using the fixed and variable collimators were within 0.1 mm.

Moreover, the MLC‐based treatment plans were generated using an FSPB algorithm, while those based on the circular collimators were developed using a ray‐tracing algorithm. Sohn et al. reported that significant discrepancies exist between the dose distributions in penumbra regions that are calculated using FSPB algorithms and measured using radiochromic film if the radiation field is small.^27^ These factors could have caused the gamma‐index passing rates to be slightly poorer for the MLC plans than for the circular collimator plans. Nevertheless, the gamma‐index passing rate exceeded 90% in each case that was investigated in this study, even in the MLC cases. According to a report by Ezzell et al.,^28^ the action level in DQA of IMRT for composite irradiation analyzed using radiographic film was 88%–90% of the gamma‐index passing rate with a criterion of 3% LPDD/3 mm DTA. In addition, Zeidan et al. stated that the percentage of dose pixels passing in GafChromic dosimetry for 10 IMRT cases, including five prostate cases, was 87% ± 8% with the 3% LPDD/3 mm DTA criterion.^29^ Hence, although it would have been preferable for the leaf position and beam modeling accuracies to have been greater in the MLC plans, it is supposed that clinically using MLCs rather than other types of collimators would not substantially impact patient outcomes. Considering the abovementioned factors, we anticipate that treatment using MLCs will be useful because of the treatment time reduction, depending on the condition of the patient, and throughput enhancement that can be realized. Therefore, an appropriate collimator type must be selected for each individual case.

In the DQA for absolute dose, the difference between the measured and calculated doses is within 3% in all cases. However, the fixed collimator results are significantly different from those obtained using the other collimator types. We confirmed that there were no steep dose gradients in the collecting volumes of the chambers in the different collimator types. However, a robust measurement point suggested by Kurosu et al.^30^ may have some effect on the results of absolute dose verification. The maximum collimator sizes of the fixed collimators were smaller than that of the other collimators. Therefore, the possibility of overlaps of small radiation fields at The PTV center that was located in the chamber could be higher.^31^, ^32^, ^33^ Due to this reason, the measurement doses of fixed collimator plans might be more influenced by the geometrical error of the chamber than that of the other collimator plans.

This study was limited in that the measurements and calculations were compared using a conventional planar DQA metric, namely, the gamma‐index passing rate. Nelms et al. demonstrated that the most common acceptance criteria and published action levels are insufficient because there is a lack of correlation between the conventional IMRT quality assurance performance metrics (e.g., the gamma‐index passing rate) and the dose differences in critical anatomical regions of interest.^34^ Therefore, to evaluate the possible clinical effects of using different types of collimators on the resulting dose distributions, patient‐specific, anatomy‐based quality assurance is required.^35^, ^36^

## 5 CONCLUSIONS

In this study, the advantages of MLCs over other types of collimators in robotic radiosurgery systems for prostrate SBRT treatment planning were clarified. Most notably, it was proven that the total MUs and treatment time can be reduced using MLCs. Moreover, although the DQA results obtained by performing film dosimetry showed that the gamma‐index passing rates resulting from using MLCs are lower than those yielded by the other types of collimators, the MLCs are sufficiently accurate for clinical use in robotic radiosurgery systems. Individual institutions must select appropriate collimator types by considering these properties.

## CONFLICT OF INTEREST

The authors declare no conflicts of interest.
